# Chordoid Glioma as a Differential Diagnosis of Anterior Third Ventricle Tumours: A Rare Case Report and Five-Year Follow-Up

**DOI:** 10.1155/2019/3584837

**Published:** 2019-12-04

**Authors:** Kristin Suetens, Jeroen Swinnen, Linde Stessens, Sofie Van Cauter, Geert Gelin

**Affiliations:** ^1^Department of Radiology, Ziekenhuis Oost-Limburg, Genk, Belgium; ^2^Department of Pathology, Ziekenhuis Oost-Limburg, Genk, Belgium

## Abstract

Chordoid glioma is a rare and relatively recently defined tumour entity. Worldwide there have only been around 90 cases described until now. A chordoid glioma comprises a low-grade suprasellar neuroepithelial neoplasm originating in the anterior part of the third ventricle, with consistent radiological features on MRI. This lesion should be considered as a differential of third ventricle tumours. The patient described in this paper is quite unique in the sense that despite only partial tumour resection was obtained, the residual tumour was not progressive during several years of follow-up. Preoperative recognition of this disease entity is crucial to modify the treatment approach and improve patient outcome.

## 1. Introduction

Chordoid glioma is a rare and relatively recently described low-grade suprasellar neuroepithelial neoplasm originating in the anterior part of the third ventricle. This entity was first named by Brat et al. in 1998 [1]. It has been classified by the World Health Organization in 2000 as a grade II. In the revised WHO classification of 2007 and 2016, this tumour has been included as a separate entity. Worldwide there have only been around 90 cases described until now.

The name of the tumour is derived from the histological appearance, resembling a chordoma, but with other characteristics pointing towards its glial origin. Most physicians have little knowledge of this tumour entity, which make it a very confusing lesion. It has to be considered as a differential of third ventricle tumours. Though it is a low-grade tumour, the treatment-related morbidity is very high. Knowledge of this disease entity is crucial to better estimate patient prognosis and improve patient outcome by modifying the treatment approach. This case report is quite unique in the sense that despite only partial tumour resection was obtained, the residual tumour was not progressive during several years of follow-up.

## 2. Case Report

A 43-year-old man presented at the neurology department complaining of increasing visual disturbances during reading, evolving into bilateral hemianopsia. In his clinical history, the patient underwent bariatric surgery one year before. Further general examination was negative.

**Figure 1:**
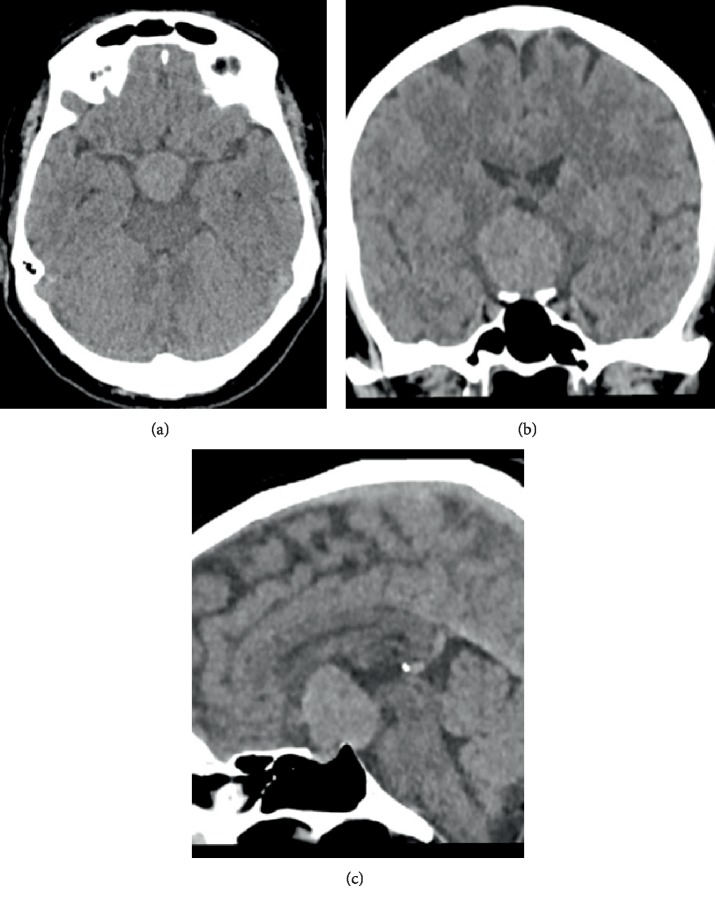
Initial contrast-enhanced brain CT. (a–c) Images show a voluminous well-circumscribed slightly hyperdense oval mass on the midline in the third ventricle, which has its largest diameter in craniocaudal direction.

First, our patient underwent a CT scan of the brain. This showed a voluminous well-circumscribed oval mass on the midline in the third ventricle, with its largest diameter in craniocaudal direction. The lesion was slightly hyperdense to the surrounding parenchyma (Figure 1).

**Figure 2:**
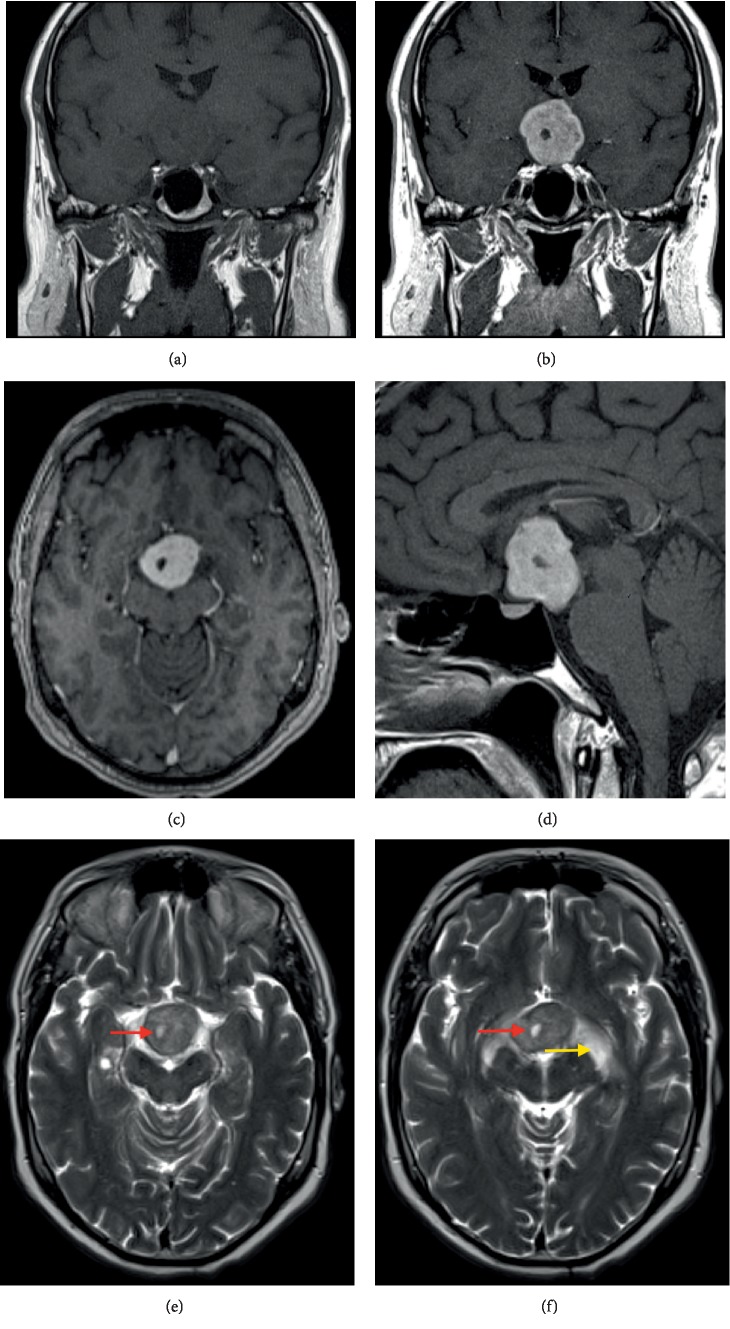
Brain MRI (a) large suprasellar mass T1-isointense to grey matter. (b–d) Vivid homogeneous T1 contrast enhancement of the lesion. (e) Axial T2-weighted images show a iso-to-hyperintense mass with a small cystic component (red arrows). (f) The mass compresses the optic chiasm, with oedematous swelling of the chiasm and both optic tracts (yellow arrow).

On the subsequent MRI of the brain, this mass was T1-isointense to grey matter, slightly heterogeneously T2-hyperintense and showed vivid homogeneous contrast enhancement after gadolinium administration (Figure 2). There was a small cystic component. The lesion compressed the optic chiasm, with secondary edematous swelling of the chiasm and the optic tracts. Furthermore, there was mass-effect on the hypothalamus and extension into the interpeduncular cistern. Biochemical screening revealed no abnormal levels of testosterone, prolactin and thyroid globulin. Of interest, this patient underwent bariatric surgery one year before, due to increasing obesity. Given the current imaging features, this was in retrospect probably part of a hypothalamic syndrome.

During surgery, a greyish mass was observed in close relation with and mass effect on the optic chiasm, which was edematous. Due to the firm consistency of the mass, only the lower part could be removed through the subfrontal access and it was decided to perform an additional transcallosal access route. A definite histopathological diagnosis could not be obtained on the frozen section material. The initial working hypothesis was a pituicytoma.

**Figure 3:**
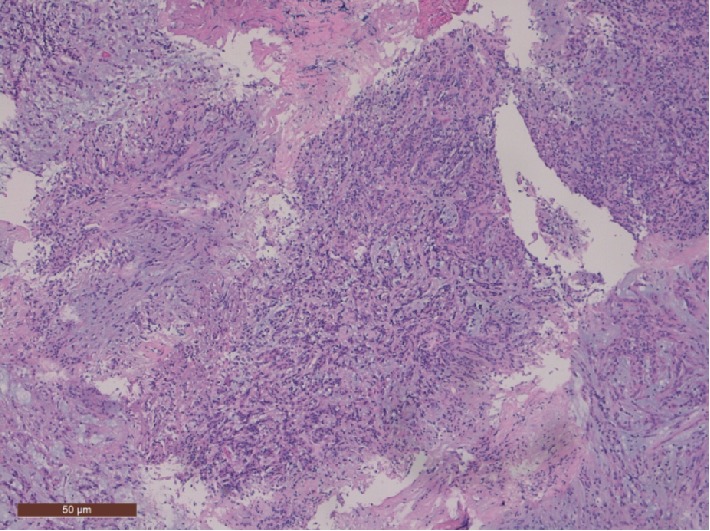
Histological features include lymphoplasmacytic infiltrate and a mucinous stroma (hematoxylin and eosin staining, original magnification ×100).

**Figure 4:**
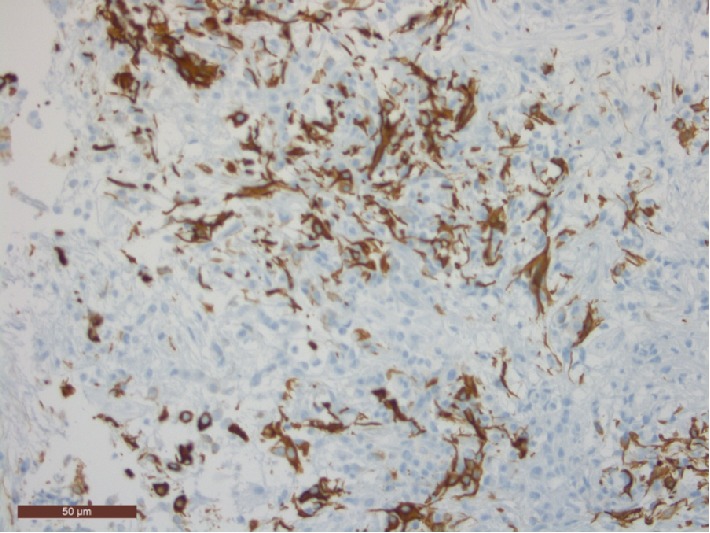
GFAP immunohistochemical staining of the tumourcells (original magnification ×400).

Detailed analysis of all available tissue material showed a dominant mucinous component with epithelial cells with eosinophilic matrix and lymphoplasmocytic infiltrate organized in clusters and cords (Figure 3). There was almost no mitotic activity. The GFAP stain was positive and there was expression of vimentin, pankeratin and EMA, and slight expression of S-100 protein (Figure 4). Analysis for thyroid transcription factor-1 (TTF-1) was not performed. Based on these findings the final diagnosis of a chordoid glioma was made.

During the postoperative hospitalization, the patient developed diabetes insipidus, for which a treatment with desmopressin was started. Later during follow-up, cortisol and thyroid deficiencies and lowered testosterone and growth hormone levels were noted, for which substitution was necessary. Furthermore, intermittent dysregulation of the ionogram with hypernatremia occurred.

Eight months later, a transcallosal re-resection of the residual tumour was planned. The patient and his family were informed about the risks and possible aggravation of the hypothalamic insufficiency. During this surgery, further debulking of the very firm tumour was performed, but complete resection was not obtained due to the tight connection with surrounding structures anteriorly and caudally.

**Figure 5:**
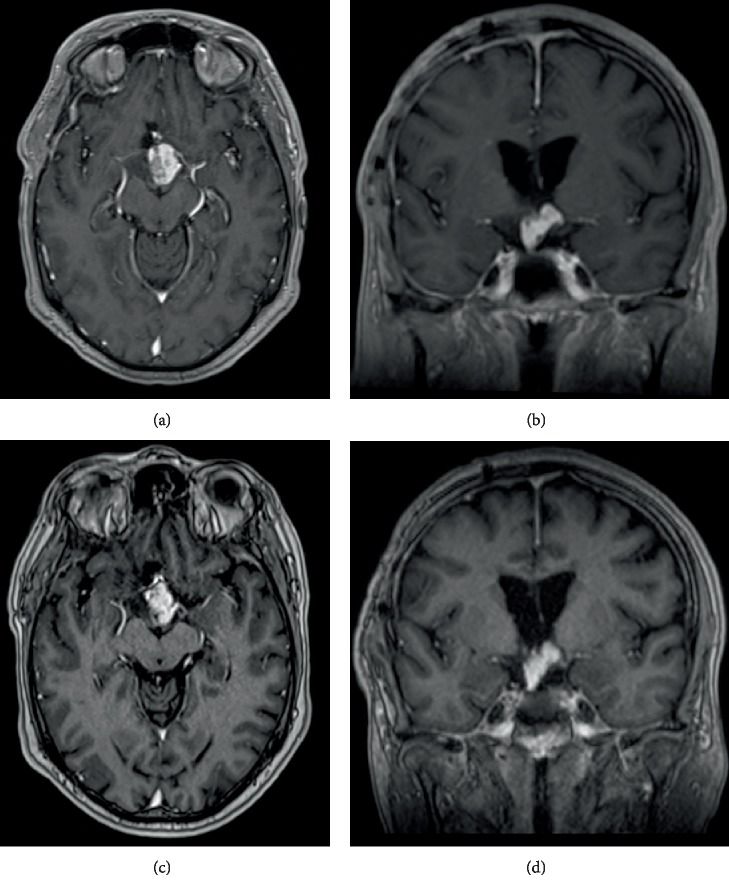
Gadolinium contrast-enhanced MRI of the brain. (a, b) First MRI after re-resection through transcallosal approach shows only partial removal of the tumour was obtained. (c, d) Follow-up study 5 years later shows no progression.

Postoperative course was complicated with severe apathy and confusion. A pronounced cognitive impairment with mainly short-term memory loss remained, for which years of revalidation were necessary with slow, but steady progression. Follow-up MRI showed no volume increase nor change in morphology during the first two years of follow-up (Figure 5). So, for that reason and because of the difficult rehabilitation, no further radiotherapy was planned. Five years later, the patient remains in close follow-up with neurological evaluation every 6 months.

## 3. Discussion

The chordoid glioma has been classified by the World Health Organization in 2000 as a grade II tumour. The name of the tumour is derived from the histological appearance, reminiscent of chordoma, but with other characteristics pointing towards its glial origin, such as avidly staining with glial fibrillary acidic protein (GFAP). In the 2007 and 2016 revisions of the WHO brain tumour classification, this tumour has been included as a separate entity. So far, relatively little is known about the genetic underpinnings of this very rare neoplasm.

The exact incidence of chordoid gliomas is not yet known, though they appear to be uncommon. They usually occur in middle-aged adults (mean age 46 years old) and have been reported more frequently in female (female : male 3 : 1) [2].

Clinical presentation varies from asymptomatic to aggressive and most often symptoms are mild and slowly progressive, with headache, amnesia and visual disturbances as most common features. Specific symptoms like endocrine alterations and visual disturbances are due to the location and its close relation to the hypothalamic and pituitary region and the optic pathways. When obstructive hydrocephalus occurs, this will give rise to more vague symptoms of increased intracranial hypertension.

The radiological features are remarkably consistent. A chordoid glioma appears as a third ventricle suprasellar mass, often in close relation to the hypothalamus.

On CT, the lesion is a well circumscribed, hyperdense (65%) mass that is homogeneously enhancing after contrast administration (68%). Most often it is oval shaped, with the greatest diameter in craniocaudal direction.

On MRI, which is the preferred examination, it appears as a slightly T2-hyperintense mass, T1-isointense to grey matter (63%), with homogeneous enhancement following contrast administration (70%). A minority of the cases (19%) show a peripheral cystic area [3]. Intralesional calcification is rare. Necrosis does not occur. The tumour can cause obstructive hydrocephalus.

Differential considerations for a vividly enhancing anterior third ventricle mass are meningioma, ependymoma, pilocytic astrocytoma, chordoma and lymphoma. This entity can sometimes be mistaken for an unruptured aneurysm as well. If there is a cystic component, a craniopharyngioma might also be suspected. Nonfunctioning pituitary tumours may be thought of, but in contrast to pituitary tumours, a chordoid glioma is distinct from the pituitary gland and stalk.

The cell of origin is unknown until now, though it has been suggested that they arise from ependymal cells (tanycytes) [2, 4, 5]. The anatomic site of origin of all reported cases can be traced to the lamina terminalis in the anterior wall of the third ventricle. The midline location may favour an embryological origin.

These tumours are described as well-circumscribed fusiform mucoid lesions. Macroscopically they consist of firm masses with a grey brownish colour [2]. Histologically they comprise clusters and cords of epithelioid cells with eosinophilic matrix and lymphoplasmacytic infiltrates, on a background of mucinous matrix. Interestingly, the vasculature lacks a blood-brain-barrier, which is consistent with the avid contrast enhancement observed in imaging studies of chordoid glioma. Immunohistochemically these tumours are diffusely and strongly positive for GFAP and vimentin, and show focal reactivity to EMA and cytokeratin in some cases, but little to no reactivity to S-100 protein.

Thyroid transcription factor-1 (TTF-1) expression has also been proposed as a good differential marker for chordoid glioma, but was not performed in our patient. In a multicentric series of 17 cases, all chordoid gliomas expressed TTF-1, as in the developing and adult lamina terminalis. Although TTF-1 is not specific to chordoid glioma, this marker could help in the differentiation of atypical cases [6, 7]. Some authors also report immunoreactivity for CD34, which is useful for making differential diagnosis between chordoid glioma, pilocytic astrocytoma and/or ependymoma. This tumour has low mitotic activity.

The chordoid glioma is a slow-growing noninvasive tumour. Complete surgical resection is considered as the treatment of choice for this type of tumour. Nonetheless, the deep location of the tumour, the close relation with the roof of the hypothalamus and the sporadic close relation to cranial nerves and the cavernous sinuses, often prevents complete resection. Intra-operative mortality rates have been reported to be 17–29% of cases, mainly due to bleeding [4, 8].

Postoperative mortality risk is highest during the first month after surgery, caused by pulmonary embolism (42%) and cardiovascular events [4]. Most patients (67%) suffer from complications after surgery, ranging from cognitive impairment to hypothalamic dysfunction [4].

Incomplete resected tumours will continue their slow growth and prompt later re-resection. Stereotactic radiosurgery has been shown to be an effective adjuvant therapy after partial resection and tends to give results superior to external beam radiotherapy.

Even though this tumour is low grade, the overall patient outcome tends to be poor, mainly owing to the location, the close proximity of critical neurovascular structures and the difficulty of obtaining a complete surgical resection. Recurrence is very rare after total macroscopic resection and metastasis is not described.

## 4. Conclusions

The chordoid glioma is a recent but distinct tumour entity. It is a rare low-grade tumour of the anterior wall of the third ventricle with consistent radiological features on MRI. Knowledge and preoperative recognition of this entity is crucial to better estimate and improve patient outcome, by modifying the treatment approach.
